# A study protocol for a feasibility trial of telephone‐delivered Adherence Therapy for adults with type 2 diabetes

**DOI:** 10.1002/nop2.735

**Published:** 2021-01-20

**Authors:** Fatimah Alenazi, Monica Peddle, Daniel Bressington, Moeber Mahzari, Richard Gray

**Affiliations:** ^1^ College of Science Health and Engineering La Trobe University Bundoora VIC Australia; ^2^ Department of Public Health College of Public Health and Health Informatics Qassim University AlBukayriyah Saudi Arabia; ^3^ Lecturer in Nursing School of Nursing and Midwifery College of Science Health and Engineering La Trobe University Melbourne Australia; ^4^ Associate Professor School of Nursing Hong Kong Polytechnic University Kowloon Hong Kong; ^5^ Adjunct Professor‐College of Nursing & Midwifery Charles Darwin University Bundoora Australia; ^6^ Assistant professor of Medicine‐Endocrinology King Saud Bin Abdulaziz University for Health Sciences College of Medicine Riyadh KSA Saudi Arabia; ^7^ Department of Medicine Division of Endocrinology – Ministry of National Guard – Health Affairs Riyadh KSA Saudi Arabia; ^8^ King Abdullah International Medical Research Center Riyadh KSA Saudi Arabia; ^9^ Professor of Clinical Nursing Practice La Trobe University Melbourne Australia

**Keywords:** diabetes, feasibility study, nurses, nursing, protocol, telephone‐delivered adherence therapy

## Abstract

**Aims:**

Adherence therapy is a candidate intervention to improve medication adherence and clinical outcomes in patients with type 2 diabetes. The feasibility of conducting a trial of adherence therapy in this population has not been established. The objective of this study is therefore to test the feasibility of conducting a randomized controlled trial of adherence therapy in a Middle Eastern context.

**Design:**

A single‐centre randomized controlled feasibility trial of adherence therapy in patients with type 2 diabetes.

**Methods:**

We will undertake an initial cultural adaptation of a telephone‐delivered form of adherence therapy in four patients in a Middle Eastern context. Our subsequent feasibility trial will aim to recruit 40 non‐adherent diabetic patients that will be randomly allocated to receive eight weekly 30‐min telephone adherence therapy sessions delivered by a diabetes educator versus treatment as usual. Key outcomes of interest include the number of patients invited to take part in the trial that consent to participate and then go on to complete treatment.

**Result:**

The findings of this study will determine the feasibility of undertaking a full randomized controlled trial of adherence therapy in patients with type 2 diabetes.

## 1 INTRODUCTION

The prevalence of diabetes mellitus is on the increase worldwide, doubling since 1980 from 4.7% of the adult population to 8.5% in 2014. The number of people living with diabetes is expected to continue to increase, affecting an estimated 550 million by 2030 and 642 million by 2040 (Ogurtsova et al., 2017).

Type 2 diabetes accounts for 90% of all diabetes cases worldwide (Ogurtsova et al., 2017). It is a long‐term condition and is associated with substantial financial burden, reduced quality of life and serious health complications including diabetic retinopathy, renal failure and heart disease (National Health Priority Action Council (NHPAC), 2006). It was estimated that five million deaths were associated with diabetic complications in 2015 (Roglic & World Health Organization [WHO], 2016). Maintaining an optimal haemoglobin A1C (HbA1c)—defined as the average blood glucose (sugar) levels for the last two to three months (Ogurtsova et al., 2017)—is a challenge for many patients. One of the biggest factors having an impact on glycaemic control is poor medication adherence (Egede et al., 2014; Pladevall et al., 2004).

## 2 BACKGROUND

A systematic review of 27 studies involving approximately 500,000 patients with type 2 diabetes reported that medication non‐adherence with oral medication alone or in combination with injectable insulin ranged from 6.9% to 61.5% (Krass et al., 2015). An earlier systematic review of 17 studies involving more than 900,000 patients with type 1 and 2 diabetes observed that non‐adherence to insulin therapy ranged from 14% to 57% (Davies et al., 2013). The considerable variation in non‐adherence rates reported in these reviews is likely due to methodological problems in measuring patient adherence to treatment.

Poor adherence to medication is an important and often overlooked barrier to optimizing health outcomes in patients with diabetes (Sokol et al., 2005) and is an important clinical issue for nurses working with this group of patients. For people with type 2 diabetes treatment, adherence is not limited to medication but also includes adopting and complying with lifestyle changes (e.g. eating a healthy diet, taking regular exercise and self‐blood glucose monitoring).

Polonsky (2016) reported that the most common modifiable factors affecting medication adherence in people with type 2 diabetes were perceived treatment efficacy, hypoglycaemia, treatment complexity and convenience, cost of treatment, medication beliefs and physician trust. Polonsky (2016) also concluded that no specific intervention has consistently been shown to be effective at improving adherence and recommended that a new approach to enhancing adherence be developed to target both treatment‐related burden and beliefs about treatment (Polonsky & Henry, 2016).

A number of different adherence interventions have been tested in patients with type 2 diabetes including patient education, consultation and simplifying treatment regimes. A Cochrane systematic review of adherence interventions in type 2 diabetes included 21 randomized controlled trials involving 4,135 patients. The authors reported that most of the studies were of methodologically poor quality and it was not possible to conclude which intervention characteristics constituted an effective medication adherence intervention (Vermeire et al., 2005).

The WHO has published guidelines regarding strategies to improve adherence to long‐term therapies that inform nursing practice. The guideline recommends supporting patients and encouraging them to participate in a treatment plan. A focus on barriers to adherence (e.g. forgetfulness), as well as addressing patients’ beliefs about their illness and treatment, is also recommended. WHO suggests that the optimum solution to non‐adherence is an individualized consultation to address patients’ belief about illness and treatment and to encourage them by actively involving patients in their treatment plan and ongoing management. As far as we can determine there have been no specific tests of this approach in patients with type 2 diabetes (Santé et al., 2003).

Many of the strategies identified by the WHO to address non‐adherence are encapsulated in adherence therapy originally described by (Gray et al., 2006). Adherence therapy is a patient‐centred approach that incorporates cognitive behavioural (e.g. problem‐solving, challenging beliefs) and motivational interviewing (exploring ambivalence) techniques to enhance medication adherence (Gray, 2011).

Adherence therapy has been tested in a number of disease areas, including mental disorders (Chien et al., 2015; Gray et al., 2006; Schulz et al., 2013), hypertension (Alhalaiqa et al., 2012) and Parkinson's disease (Daley et al., 2014). For example, a randomized controlled trial of seven adherence therapy sessions facilitated by nurses versus treatment as usual was conducted with 136 non‐adherent patients with hypertension, resulting in significant improvements in adherence to treatment in the intervention group compared with the control group (Alhalaiqa et al., 2012). Similarly, Daley et.al (2014) tested adherence therapy facilitated by nurse specialists in a randomized controlled trial of 76 non‐adherent patients with Parkinson's disease. Improvements in both medication adherence and quality of life were reported by the patients who received adherence therapy (Daley et al., 2014). A systematic review and meta‐analysis of six randomized controlled trials involving 725 patients also showed that adherence therapy was effective at improving psychiatric symptoms in patients with severe mental illness with a medium effect size (SMD = 0.56). However, adherence behaviours and treatment attitudes did not improve significantly (SMDs of 0.25 and 0.43, respectively) (Gray et al., 2016). Importantly, research outcomes indicate that adherence therapy could potentially be facilitated by nurses as part of their routine clinical practice (Gray et al., 2004).

Whilst there may be merit to undertaking a trial of adherence therapy in patients with type 2 diabetes, consideration needs to be given as to how adherence therapy can be delivered as part of routine nursing practice as patients visit secondary care diabetes clinics infrequently (e.g. six monthly). Mobile telephone usage has become pervasive since the early 2000s and is a potentially novel way to deliver psychological interventions. Telephone‐delivered interventions may be preferred by patients because the interventions consume less time (e.g. there is no travel to and from a hospital) and can be more easily delivered at a time that fits in with their lifestyle (e.g. outside of normal clinic hours). Moreover, systematic reviews and meta‐analyses have reported that it is feasible to deliver psychological interventions over the phone (Fjeldsoe et al., 2009; Krishna & Boren, 2008; Liang et al., 2011).

The feasibility of conducting a trial adherence therapy trial in a Middle Eastern context is the research question that this study will address. A feasibility trial is a study undertaken to inform a main clinical trial. Important parameters that will inform main trial design can be tested. Typically, these include participants willingness to be randomized, subjects motivation to consent to take part in a trial and an estimated of the number of patients the meet eligibility criteria (Arain et al., 2010). In nursing science, this stage in the research process is often omitted substantially increasing the risk that a full trial will not recruit to time and target (Gray et al., 2019).

## 3 THE STUDY

### 3.1 Design

The study is in two parts: 1. a qualitative cultural adaptation, 2. a parallel group, unblinded, randomized controlled feasibility trial.

## 4 METHODS

### 4.1 Part one: Cultural adaptation

Adherence therapy has not been previously implemented in diabetes patients in a Middle Eastern context. To establish the acceptability of the therapy in the Middle East and to identify any required modifications to the intervention, a cultural adaption study will be completed prior to undertaking the feasibility trial. The study will explore patients’ willingness to actively participate in a therapy process that is focused on them reflecting on how best to manage their diabetes when the typical cultural expectation is for health professionals to be wholly responsible for managing the patient's illness (Chamsi‐Pasha & Albar, 2016). Most Middle Eastern countries have a strong Islamic tradition that likely has an impact on health beliefs. A review of 34 studies investigating determinants of patients’ behaviour in Middle Eastern countries showed that culture including lifestyle, self‐beliefs, religions and healthcare providers trust effect the management of type 2 diabetes. In Muslim society, religion plays an important role in how clinical care is delivered. For example, death and sickness are in God's plan and we have no control on it (Alsairafi et al., 2016). This may lead to a sense of passivity, whereby the individual does not think they have control or influence over their illness.

### 4.2 Transcultural experience of the research team

The practical application of an intervention depends heavily on addressing patients’ beliefs and culture (Marsiglia & Booth, 2015). The research team has considerable transcultural experience. Two members of the team are from Saudi Arabia: MM is a consultant endocrinologist and FA a certified diabetes educator. RG worked for three years in the national health system of a Middle Eastern country, and DB has worked in Asia for the past five years. MP coordinates and teaches transnational education programmes in Asia.

### 4.3 Approach to cultural adaptation

#### 4.3.1 Population

Four adults with type 2 diabetes from the National Guard Hospital in Riyadh, Saudi Arabia will be recruited. Patients will be approached by a member of the treating team and asked if they are interested in participating in the cultural adaptation study. If they agree, they will be asked for their contact information (phone number) to be contacted by the researcher and verbal consent to approach will be documented in the patients’ electronic medical record. The researcher will then discuss the study and answer any questions they might have. If patients indicate that they want to take part, FA will send a patient information sheet describing the project and electronic consent (using the REDCAP eConsent procedure) will be obtained.

#### 4.3.2 Intervention

Participants will receive their usual care plus eight sessions of adjunctive telephone adherence therapy delivered twice weekly for 4 weeks. Each session will last approximately 30 min. FA will deliver adherence therapy under the supervision of RG and DB.

#### 4.3.3 Data collection

Before participating in the study, an online baseline assessment questionnaire will be sent. The questionnaire will consist of demographic information, beliefs about medication and medication adherence. Also, included in the demographic information will be questions about their current medication regimen and their most recent HbA1c level. To check the accuracy of self‐reporting, participants answers will be checked with the details of prescribed medication provided in the medical records. The aim of the baseline assessment is to check the validity of self‐reporting and if an online questionnaire is appropriate for this patient group. FA will also interview participants at the end of therapy. The interview will be via phone, last approximately 30 min and will focus on our approach to recruitment, the appropriateness of the measures and the acceptability of the therapy. Interviews will be transcribed and subject to thematic analysis using the approach originally described by Braun and Clark (Braun & Clarke, 2006).

#### 4.3.4 Findings

The research team will meet to consider any changes that need to be made based on our findings. Any changes that may be required to the feasibility trial methodology as a consequence of this process will be submitted to the appropriate ethical committee and trial steering committee for review.

### 4.4 Part two: Feasibility trial

This study is a parallel‐group open‐label trial to test the feasibility of conducting a full randomized control trial of adherence therapy in patients with type 2 diabetes in a Middle Eastern context. According to the National Institute for Health Research (NIHR), a feasibly study is designed to check if the main randomized control trial can be completed. Some of the possible objectives of a feasibility trial are to estimate the number of eligible patients and the likely recruitment rate (NIHR, 2019). This protocol adheres to the SPIRIT guidelines (A.‐W. Chan et al., 2013) for clinical trial protocols.

### 4.5 Objectives

In this trial, we aim to establish whether patients:

1. Can be recruited to the trial.
2. Complete treatment (at least five out of eight sessions) with telephone‐delivered adherence therapy.
3. Complete study measures using an online web‐based data entry system (RedCAP).

### 4.6 Study setting

The study will take a place at the National Guard Hospital, Riyadh, Kingdom of Saudi Arabia. The hospital is one of the largest hospitals in Saudi Arabia with 1,505 beds employing 4,000 nurses and 1,600 physicians. The hospital provides inpatient and outpatient services primarily to people who work for the Saudi Arabian National Guard and their families. A full complement of tertiary services are also provided to the wider regional population (Division of Endocrinology, n.d.).

Participants will be recruited from the diabetes outpatient clinic. The clinic is led by a group of consultants, nurses and other clinicians who assess and review patients and amend their medication or treatment as required. Diabetes educators, predominantly nurses and dietitians, meet with patients to review their medication and provide diabetes education. Patient education is essentially instructional in nature; patients are given factual information by the educators. There is minimal opportunity for discussion between the educator and patient. The clinical service sees around 100 patients with type 2 diabetes a week.

### 4.7 Eligibility criteria

Patients will be eligible to participate in this trial if they meet the following criteria.

#### 4.7.1 Inclusion criteria

- Adults over the age of 18 years.
- Diagnosed with type 2 diabetes for at least one year.
- Prescribed any oral or self‐administered injectable medication for the treatment of diabetes (e.g. metformin, insulin).
- Are non‐adherent to prescribed medication (defined as a Medication Adherence Reporting Scale [MARS] score < 5).

#### 4.7.2 Exclusion criteria

- Are participating in any other clinical trial.
- Are currently receiving any other psychological or psychosocial intervention (either face‐to‐face, telephone or online) as this may contaminate treatment effects.
- Have a terminal illness likely to lead to death within the study period.
- Have any of the following long‐term conditions: brain injury, psychosis, dementia, cognitive impairment or memory problems. We are excluding these patients as these co‐morbid conditions may make it difficult for the patients to engage in treatment.
- Are hearing impaired, as the programme is delivered via phone and requires the participants to communicate verbally.

### 4.8 Interventions

#### 4.8.1 Treatment as usual

Participants in the control and experimental groups will continue receiving their usual care. Treatment as usual for adults with type 2 diabetes includes regular doctor visits and meeting with the health educator if referred by the doctor. Meeting with the diabetes educator includes reviewing the patient's glycaemic control tests, glucose monitoring and a 20‐min didactic educational session.

#### 4.8.2 Adherence therapy

A manualized intervention, adherence therapy aims to enhance medication adherence by discussing and carefully challenging patients’ beliefs about medication (e.g. I don't need to take medication if I have no symptoms) and exploring ambivalence about taking medication for diabetes (Gray, 2011). There is also a focus on addressing practical problems (e.g. I forget to take medication) in the adherence therapy approach (Alhalaiqa et al., 2013; Gray et al., 2016). Participants receiving adherence therapy will be sent a SMS about the date/time of the session a day in advance.

In this trial, adherence therapy will be delivered over the phone by FA, a Saudi diabetes educator who trained at the National Guard Hospital. FA will be trained to deliver adherence therapy by RG who initially described the intervention. Training will include a combination of online courses and weekly one‐hour training sessions with RG. In total, FA will attend 12 hr of training. FA will also receive fortnightly supervision from RG and/or DB.

To determine fidelity with adherence therapy, a sample of ten randomly selected sessions will be audio recorded and rated using the Adherence Therapy Scale (ATS) (Gray et al., 2006). Participants will indicate on the consent form if they are willing to have sessions recorded. Not agreeing to have the session audio recorded will not preclude people from participating in the trial.

### 4.9 Outcomes

#### 4.9.1 Primary outcome (feasibility)

The primary outcome of this trial is the feasibility of recruiting people diagnosed with type 2 diabetes to adherence therapy trial in a Middle Eastern context. Our feasibility outcomes are, therefore, the number of patients:

- Asked about the study who express an interest in participating and request further information.
- That need to be asked to get one to provide informed consent.
- That complete baseline measures.
- Who complete treatment (defined as attending five of eight sessions with a session being defined as talking on the phone for at least ten minutes).
- Who complete postintervention measures.

#### 4.9.2 Secondary outcomes (planned outcome measures for the trial)

We will summarize quantitative data using descriptive statistics (mean, standard deviation, 95% CI) outcome measures. They are as follows:

- Adherence determined using the MARS.
- Self‐reported HbA1c.
- Beliefs about medication, determined using the BMQ.
- The frequency of missing items across all the measures.

### 4.10 List of measures

#### Demographics data

The following demographic information will be collected: age (in years), gender (male, female, other), country of birth, marital status, number of children, highest level of education, city of residence, employment status, family income, duration of diabetes and number of medical comorbidities.

#### Patient's adherence

To determine patient adherence, we will use the Medication Adherence Reporting Scale (MARS‐5) that was initially developed and validated by (A. H. Y. Chan et al., 2020). The questionnaire has five Likert scale items, for each item answer categories ranging from 1 least adherent to 5 most adherent. The MARS scale was translated and validated in Arabic (Alsous et al., 2017) and has been used to determine adherence in patients with diabetes (Borgsteede et al., 2011; de Vries et al., 2014).

#### Patient's beliefs about medication

Patient's beliefs about medicine will be measured using the Beliefs about Medication Questionnaire (BMQ) (Horne et al., 1999). The BMQ is a validated questionnaire that measures beliefs relating to prescribed medication and the patients general view about taking medication. The questionnaire has been translated and validated into Arabic by (Alhalaiqa et al., 2015) and used to measure patients with diabetes beliefs about medication (Schoenthaler et al., 2012; de Vries et al., 2014).

#### 4.10.1 Participant timeline

Initial patient screening and consent to approach will be undertaken by the treating team when the patients attend for routine hospital appointments. The researcher will explain the study and obtain online consent within 2 weeks of consent to be approached being obtained (week one). Baseline measures (week two) will be completed within a week of online consent being obtained. Participants will be randomized and informed of group allocation by FA within 2 days of baseline measures being completed. In the intervention group, patients will receive at least weekly sessions of adherence therapy (week three through 12). The intervention will be delivered by telephone and will be no more than 30 min in duration. Treatment will be finished within 10 weeks of the baseline assessment being completed. Patients will be defined as treatment completers if they attend at least five sessions. A completed session is defined as being of at least ten minutes in duration. Qualitative interviews and follow‐up assessments (study end point) will be completed between weeks 13 and 14. The draft CONSORT flow chart can be accessed via Figshare: https://latrobe.figshare.com/articles/CONSORT_feasibility_randomised_controlled_trial_of_adherence_therapy_in_type_two_diabetes_pdf/9724133

#### 4.10.2 Sample size

This study will not evaluate the efficacy of adherence therapy; consequently, a formal sample size calculation is not required. There is no clear convention about the number of participants required in a feasibility trial. However, a number of authors have stated that at least 30 participants are required to draw reasonable conclusions about the feasibility of conducting a full trial (Arain et al., 2010; Julious, 2005). Therefore, the sample size in our trial will be 40 patients with a 1:1 randomization ratio.

#### 4.10.3 Recruitment

Recruitment of participants will be undertaken at the outpatient clinic of the National Guard Hospital. Approximately 100 patients with type 2 diabetes are seen each week. We estimate that approximately 50% of patients will be non‐adherent according to our definition (i.e. MARS score ≥ 5). In previous adherence studies, around six patients need to be asked to get one to consent (Chien et al., 2015). This means that we can realistically expect to recruit around four patients per week to the trial. Recruitment can be expected to be completed within 5 weeks. Our recruitment will follow the following process:

- Step one (checking eligibility): the treating team working in the diabetes clinic will have access to a list of patients who will be seen in the clinic each day. They will undertake an initial screen (i.e. determine they are over 18 years of age and have a diagnosis of type 2 diabetes).
- Step two (consent to approach): a member of the treating team will then ask patients if they would be interested in taking part in a research project about adherence to diabetes treatment (consent to approach). If they say yes, they will be given a patient information sheet and ask the patient to complete the MARS. At this time, patients will also be told that only patients who regularly miss their medication will be included in the study. Patients who score below seven on the MARS will be informed they meet inclusion criteria and be asked to provide consent. Adherent patients (i.e. those with a MARS score above eight) will be informed that “because adherence does not seem to be a problem for them, they may not benefit from the intervention and on this occasion cannot be included.” Information about the number of people who receive study information and who consent to be approached will be recorded.
- Step three (informed consent): from patients that meet study inclusion criteria, we will seek online informed consent. FA will explain study procedures and answer any questions participants may have. Participants will have 48 hr to consider taking part in the study at which point online informed consent will be obtained.

#### 4.10.4 Allocation

To ensure allocation sequence is random and concealed, we will use an external randomization service (www.sealedenvelope.com ) for this trial. Sealed Envelope uses a computer‐generated random sequence and randomly permuted blocks of four, six or eight (Sealed Envelope Ltd., 2019). Allocation will be on a 1:1 ratio (adherence therapy or treatment as usual).

#### 4.10.5 Blinding

This is an open‐label study. Both the participants and the researcher will be aware of group allocation. Because this is a feasibility trial, the risk of bias that this may introduce is not of particular concern.

### 4.11 Data collection methods

#### Feasibility outcomes for this trial are the number of participants

In addition to the feasibly outcomes, we will record the following in the adherence therapy group:

- Total duration (in minutes) of treatment.
- Length of each session (in minutes).
- Number of sessions (defined as at least a ten‐minute discussion) attended.
- Number of sessions cancelled.
- Number of sessions rescheduled.

#### Qualitative interviews

All participants will be invited to participate in follow‐up interviews after completing the weeks 12–14 follow‐up assessment. Participants from both groups will be asked about their views of being in the trial. Additionally, participants in the intervention group will be asked about the usefulness of adherence therapy, the mode of delivery and satisfaction with the experimental intervention.

### 4.12 Data management

#### Data forms and security

REDCap will be adopted as an electronic Case Record Form (eCRF). REDCap was specifically designed as a secure internet‐based eCRF and has been programmed so that participants cannot make errors in data entry. For example, participants will only be able to enter age as a two‐digit numerical field. If the participant tried entering their age as 200—by adding an additional 0—they would not be able to do so. Conversely, if they entered their age as 2—by missing a 0—this would also be rejected, and the participant would be asked to enter a valid age. Participants will need to complete every field and will not be able to proceed from one question to the next unless they do so. That said, it is ethically important that participants have the option to not answer questions (eg, about gender) should they choose. Participants will be sent a link—by both SMS and email—explaining that they need to complete the study questionnaires online. They will also be informed that if they require assistance in completing the questionnaire, they can telephone the study researcher who will assist them.

#### Process of selecting sessions to audio record

Ten adherence therapy sessions, chosen at random, will be audio recorded, transcribed and rated using the Adherence Therapy Checklist (fidelity measure) (Gray et al., 2006). The process of randomly selecting sessions for recording will be as follows. Patients will be assigned session number (e.g. patient one will be assigned session one through eight; patient two, nine through 16 etc.). We will use a random number generator to put the 160 sessions into a random order. The researcher will select the first ten sessions. When these sessions occur, the researcher will check consent with the participant and audio record the session. If the patient declines, the researcher will move to the next session on the list until ten sessions have been recorded.

#### Recording of qualitative interviews

Participants in both groups will be interviewed between weeks 12 and 14. The purpose of the interview will be to elicit participants’ feedback about the acceptability of trial procedures and adherence therapy (intervention group only). A smartphone (only used for the trial and disconnected to the internet) will be used for audio recording interviews. Immediately following the interview, the audio file will be uploaded to the University research drive and deleted from the smartphone. Interviews will be transcribed by the researchers (in Arabic) and sent to participants for checking. Any identifiable information will be removed from the transcripts.

#### 4.12.1 Analysis

We will report summary statistics (number, percentage) against each of the study aims (e.g., *N* = X patients expressed an interest in participating in the study of which *N* = X (x%) consented to participate).

##### Qualitative data analysis

Transcripts will be translated from Arabic to English before coding and analysis. The translation will be done using two steps of translation: forward and backward by two bilingual persons. FA will translate the original Arabic transcripts into English, and then, another bilingual translator will do the backward translation. The two bilingual persons will then agree on a final English version of each transcript.

Qualitative data will be analysed using inductive approaches within thematic analysis following the six steps described by Braun and Clarke (Braun & Clarke, 2006) with themes and codes driven by the data. The steps start with the researchers becoming familiar with the data by reading the transcripts and generating initial ideas from the data to create codes. Two researchers will read the transcripts independently and code the data. Identified codes will be discussed for coherency, consistency and fit with the research group with modifications made by consensus of the group. Identified codes will be combined to create themes and subthemes. Each theme will then be identified and defined, and a thematic map will be generated.

### 4.13 Data monitoring

#### Data safety and monitoring board (DSMB)

Because this is a clinical trial that does not involve a medicinal product, a DSMB is not required.

#### Harms

In our trial, we will adopt the Good Clinical Practice (GCP) definition of adverse events, that is “any untoward medical occurrence in a patient or clinical investigation subject […] which does not necessarily have a causal relationship with this treatment.” The researcher will monitor for any adverse events that have occurred during the study period. Safety reporting will comply with the Australian Clinical Trial Handbook ("Australian Clinical Trial Handbook", 2018).

#### 4.13.1 Ethics and protocol amendments

Patients in this study will be recruited from the National Guard Hospital in Riyadh. Ethics approval has been obtained from King Abdullah International Medical Research Centre (KMARC) (reference number: RYD‐19–419812–147869) and La Trobe University Human Research Ethics Committee (Reference number: HEC19221). Any changes to the trial protocol will require a formal amendment to the protocol from all the respective parities the LTU ethics committee and the Saudi ethics committee (King Abdullah International Medical Research Centre (KMARC)).

#### 4.13.2 Governance

All of the investigators have a current Good Clinical Practice certificates. An independently chaired trial steering committee (TSC) reporting to the School Research Committee will have oversight of the conduct of the trial. The committee will meet 4 times during the trial. FA will report recruitment against target and any adverse events that occur during the trial. Patients who suffer from adverse events during our trial will have access to the National Guard Hospital emergency room and can contact MM to schedule appointment for consultation. This has been included in the consent form. The data will be stored on La Trobe University research servers. An anonymized data set will be published with the results manuscript.

## 5 DISCUSSION

### 5.1 Potential study implications

Previous systematic reviews indicate that there is no clear evidence of an ideal therapy or intervention that helps patients with type 2 diabetes improve their medication adherence (Nieuwlaat et al., 2014; Williams et al., 2014). Therefore, a new intervention method and innovation is necessary to improve medication adherence. Adherence therapy is an interesting potential candidate intervention in patients with type 2 diabetes. As far as we can determine there have been no previous trials of telephone‐delivered adherence therapy in patients with type 2 diabetes in a Middle Eastern context. We therefore propose a two‐part study including, first, a cultural adaptation study followed by a feasibility trial to test telephone‐delivered adherence therapy in patients with type 2 diabetes.

### 5.2 Limitations

The researcher is delivering the therapy and collecting outcome data. This may introduce the potential for bias and coercion. Also, participants will not be blinded in this study. Some of the medical information such as HbA1c will be self‐reported. This feasibility study does not have the power to test differences between groups.

### 5.3 Conclusion

- Poor adherence to diabetes treatment is an important public health issue.
- Adherence therapy is a promising intervention that has not been applied in people with type 2 diabetes in Middle Eastern context.
- We will undertake a transcultural adaptation of adherence therapy in a Middle Eastern context.
- This study will establish if adherence therapy delivered via telephone is an acceptable intervention for patients with type 2 diabetes.
- The feasibility of conducting a full randomized controlled trial to test the effectiveness of adherence therapy in patients with type 2 diabetes will be established in this study.
- This protocol adheres to the SPIRIT guidelines for clinical trial protocols and was prospectively registered with the Australian and New Zealand Clinical Trials Registry.
- This study will establish if adherence therapy is an intervention that may be incorporated into the practice of nurses working with people with diabetes.

## 6 TRIAL REGISTRATION

The trial protocol was prospectively registered in the Australian New Zealand Clinical Trials Registry (ANZCTR) on the 07/06/2019 trial ID: ACTRN12619000827134.

## CONFLICTS OF INTEREST

RG has developed adherence therapy but has no financial benefit from the therapy or the trial. FA, MM, DB and MP declare that there is no conflict of interests to declare.

## AUTHOR CONTRIBUTIONS

RG and FA conceived the trial. DB, MM and MP contributed to revisions to the study design. FA wrote the first draft of the manuscript. All authors contributed revisions to the paper. All authors approved the final version of the protocol prior to submission for publication.

## Data Availability

As this is a protocol, there are no data to present.
